# Supplementary material for: Phenotyping the Histopathological Subtypes of Non-Small-Cell Lung Carcinoma: How Beneficial Is Radiomics?
Source: Diagnostics (Basel). 2023 Mar 18;13(6):1167. doi: 10.3390/diagnostics13061167 (PMC10046953; doi:10.3390/diagnostics13061167)
Supplement: Supplementary file 1 [file diagnostics-13-01167-s001.zip › diagnostics-2239766-supplementary.pdf]

Supplementary Material

Batch analysis and feature harmonization

Kruskal-Wallis ANOVA Table

Source	SS	df	MS	Chi-sq	Prob>Chi-sq
Groups	2.30853e+06	2	1154263.7	127.3	2.28089e-28
Error	6.12433e+06	463	13227.5		
Total	8.43285e+06	465			

Kruskal-Wallis ANOVA Table

Source	SS	df	MS	Chi-sq	Prob>Chi-sq
Groups	3.51784e+06	2	1758919.8	193.98	7.55124e-43
Error	4.91501e+06	463	10615.6		
Total	8.43285e+06	465			

Figure S1. Kruskal-Wallis for PC1 on top, Kruskal-Wallis for PC2 on the bottom for the 4-classes dataset.

Kruskal-Wallis ANOVA Table

Source	SS	df	MS	Chi-sq	Prob>Chi-sq
Groups	1.86791e+06	2	933957.3	134.32	6.78947e-30
Error	3.79183e+06	405	9362.5		
Total	5.65974e+06	407			

Kruskal-Wallis ANOVA Table

Source	SS	df	MS	Chi-sq	Prob>Chi-sq
Groups	2.19839e+06	2	1099196.11	158.09	4.69145e-35
Error	3.46135e+06	405	8546.54		
Total	5.65974e+06	407			

Figure S2. Kruskal-Wallis for PC1 on top, Kruskal-Wallis for PC2 on the bottom for the 3-classes dataset.

Kruskal-Wallis ANOVA Table

Source	SS	df	MS	Chi-sq	Prob>Chi-sq
Groups	884981.9	2	442491	116.06	6.29286e-26
Error	1410293.6	299	4716.7		
Total	2295275.5	301			

Kruskal-Wallis ANOVA Table

Source	SS	df	MS	Chi-sq	Prob>Chi-sq
Groups	773578.7	2	386789.3	101.45	9.35877e-23
Error	1521696.8	299	5089.3		
Total	2295275.5	301			

Figure S3. Kruskal-Wallis for PC1 on top, Kruskal-Wallis for PC2 on the bottom for the 2-classes dataset.

Kruskal-Wallis ANOVA Table

Source	SS	df	MS	Chi-sq	Prob>Chi-sq
Groups	40110.6	1	40110.6	2.21	0.137
Error	8392741.9	464	18087.8		
Total	8432852.5	465			

Kruskal-Wallis ANOVA Table

Source	SS	df	MS	Chi-sq	Prob>Chi-sq
Groups	36.4632	1	36.5	0	0.9642
Error	8432816.0368	464	18174.2		
Total	8432852.5	465			

Figure S4. Kruskal-Wallis for PC1 on top, Kruskal-Wallis for PC2 on the bottom after harmonization for the 4-classes dataset.

Kruskal-Wallis ANOVA Table

Source	SS	df	MS	Chi-sq	Prob>Chi-sq
Groups	33742.5	1	33742.5	2.43	0.1193
Error	5625999.5	406	13857.1		
Total	5659742	407			

Kruskal-Wallis ANOVA Table

Source	SS	df	MS	Chi-sq	Prob>Chi-sq
Groups	1045.24	1	1045.2	0.08	0.784
Error	5658696.76	406	13937.7		
Total	5659742	407			

Figure S5. Kruskal-Wallis for PC1 on top, Kruskal-Wallis for PC2 on the bottom after harmonization for the 3-classes dataset.

Kruskal-Wallis ANOVA Table

Source	SS	df	MS	Chi-sq	Prob>Chi-sq
Groups	14597.7	1	14597.7	1.91	0.1665
Error	2280677.8	300	7602.3		
Total	2295275.5	301			

Kruskal-Wallis ANOVA Table

Source	SS	df	MS	Chi-sq	Prob>Chi-sq
Groups	5485.27	1	5485.27	0.72	0.3964
Error	2289790.23	300	7632.63		
Total	2295275.5	301			

Figure S6. Kruskal-Wallis for PC1 on top, Kruskal-Wallis for PC2 on the bottom after harmonization for the 2-classes dataset.

Feature selection and stability

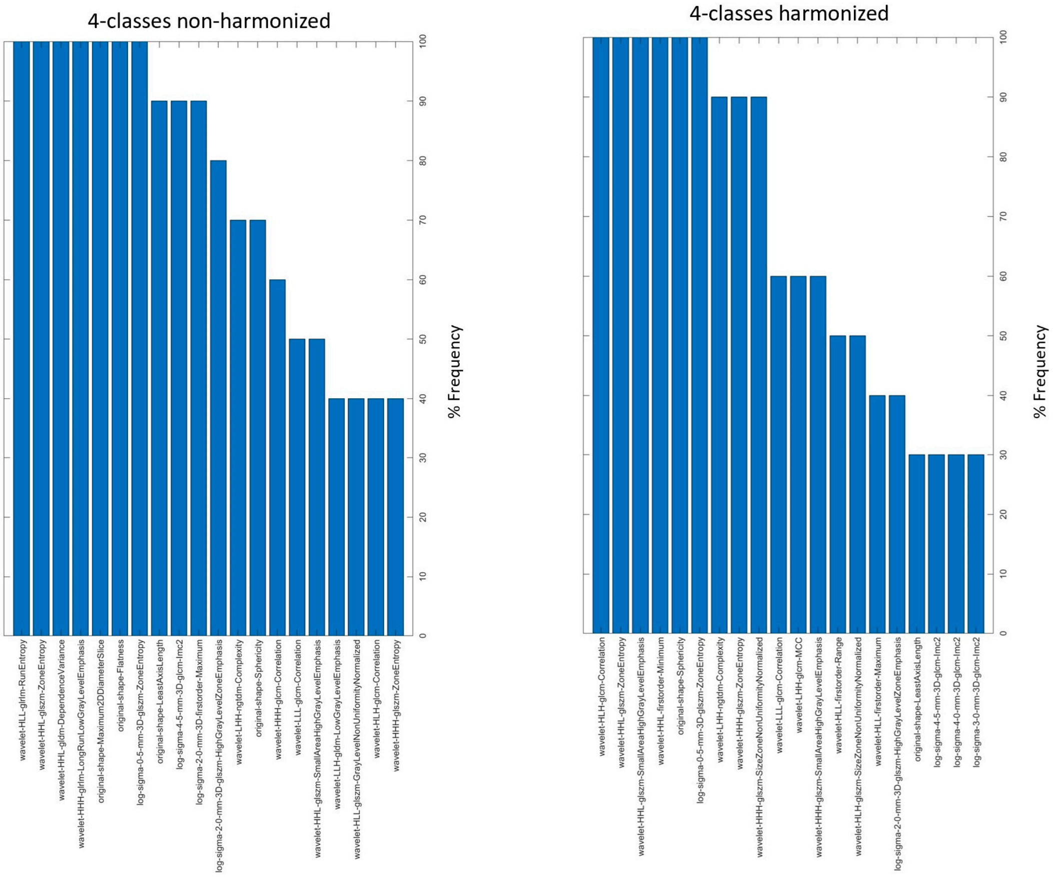

Figure S7. Feature stability for the 4-classes datasets

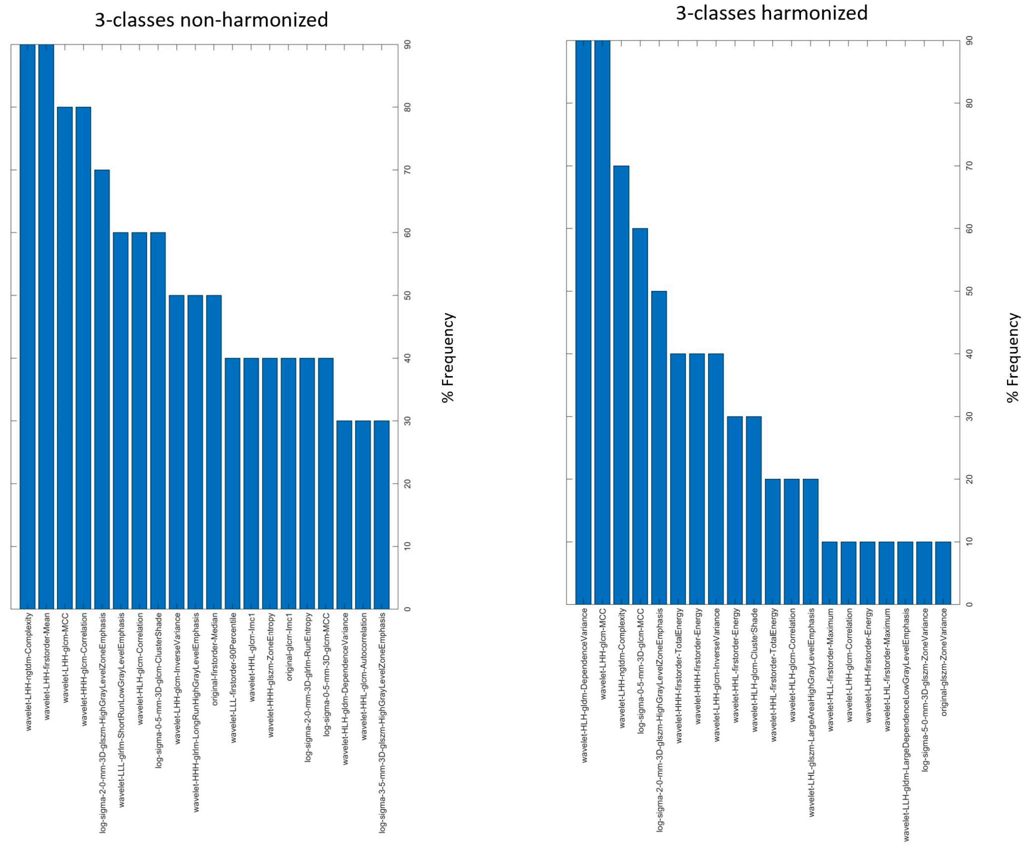

Figure S8. Feature stability for the 3-classes datasets

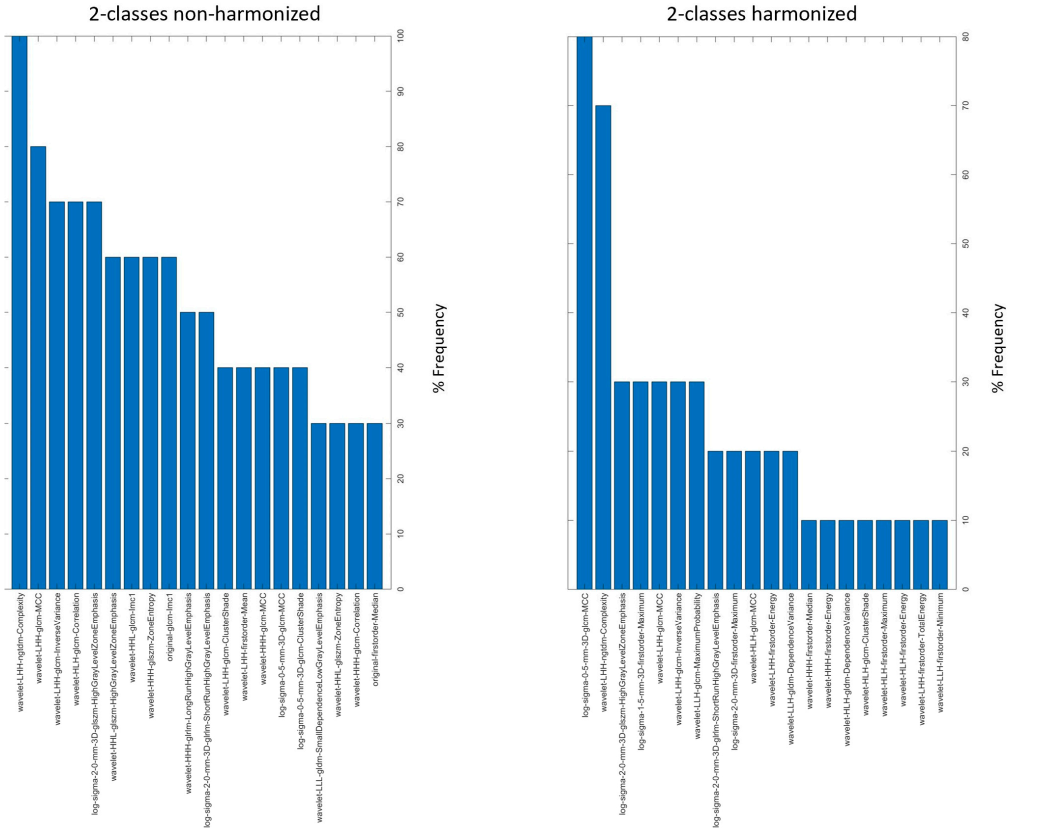

Figure S9. Feature stability for the 2-classes datasets

Classification

Table S1. 4-classes dataset, **SVM: test accuracy = 0.6141±0.0317**

	auc	tpr	tnr	ppv	fscore
NOS val	0.9614±0.0090	0.8908±0.0211	0.9819±0.0022	0.8770±0.0142	0.8830±0.0146
NOS test	**0.9608±0.0325**	**0.8981±0.0546**	**0.9843±0.0088**	**0.8940±0.0585**	**0.8933±0.0162**
ADC val	0.8120±0.0095	0.6912±0.0173	0.8688±0.0103	0.7168±0.0180	0.7026±0.0209
ADC test	0.8225±0.0365	0.7059±0.0615	0.8783±0.0269	0.7404±0.0554	0.7205±0.0097
LCC val	0.8015±0.0094	0.4264±0.0201	0.8517±0.0069	0.4513±0.0265	0.4268±0.0492
LCC test	0.7866±0.0331	0.4103±0.0650	0.8404±0.0188	0.4173±0.0438	0.4037±0.0540
SCC val	0.7403±0.0063	0.5789±0.0157	0.7608±0.0107	0.5417±0.0102	0.5575±0.0504
SCC test	0.7233±0.0287	0.5588±0.0469	0.7520±0.0329	0.5247±0.0395	0.5378±0.0387

The highest values of interest are highlighted in bold.

Table S2. 4-classes dataset harmonized, **Ensemble: test accuracy = 0.5624±0.0555**.

	auc	tpr	tnr	ppv	fscore
NOS val	0.9698±0.0075	0.9061±0.0107	0.9950±0.0016	0.9651±0.0109	0.9340±0.0091
NOS test	**0.9573±0.0265**	**0.8848±0.0355**	**0.9972±0.0023**	**0.9793±0.0161**	**0.9281±0.0199**
ADC val	0.8106±0.0132	0.6665±0.0243	0.8164±0.0127	0.6332±0.0178	0.6484±0.0177
ADC test	0.7995±0.0494	0.6458±0.0701	0.8072±0.0354	0.6205±0.0627	0.6303±0.0621
LCC val	0.7559±0.0120	0.3614±0.0411	0.8727±0.0175	0.4549±0.0240	0.3902±0.0305
LCC test	0.7296±0.0268	0.3128±0.0779	0.8550±0.0367	0.3760±0.0488	0.3255±0.0572
SCC val	0.7224±0.0125	0.5677±0.0234	0.7365±0.0183	0.5141±0.0171	0.5360±0.0162
SCC test	0.6913±0.0360	0.5344±0.0594	0.7137±0.0424	0.4800±0.0440	0.4996±0.0432

The highest values of interest are highlighted in bold.

Table S3. 3-classes dataset, **KNN: test accuracy = 0.6027±0.0347**

	auc	tpr	tnr	ppv	fscore
ADC val	0.8335±0.0100	0.7173±0.0096	0.8786±0.0102	0.7752±0.0148	0.7446±0.0100
ADC test	**0.8519±0.0237**	**0.7390±0.0292**	**0.8814±0.0284**	**0.7900±0.0387**	**0.7616±0.0226**
LCC val	0.7684±0.0191	0.4791±0.0827	0.8196±0.0235	0.4815±0.0260	0.4677±0.0534
LCC test	0.7670±0.0371	0.4652±0.0713	0.8093±0.0652	0.4873±0.0782	0.4575±0.0493
SCC val	0.7041±0.0214	0.5981±0.0298	0.7148±0.0458	0.5623±0.0342	0.5752±0.0160
SCC test	0.6855±0.0404	0.5627±0.0962	0.7120±0.0316	0.5311±0.0399	0.5408±0.0667

The highest values of interest are highlighted in bold.

Table S4. 3-classes dataset harmonized, **Ensemble: test accuracy = 0.5295±0.0555**

	auc	tpr	tnr	ppv	fscore
ADC val	0.7501±0.0557	0.6374±0.0358	0.7896±0.0680	0.6492±0.0752	0.6407±0.0541
ADC test	**0.7432±0.0616**	**0.6173±0.0656**	0.7837±0.0627	**0.6371±0.0870**	**0.6234±0.0723**
LCC val	0.6947±0.0648	0.3864±0.1118	**0.8389±0.0445**	0.4122±0.1069	0.3913±0.1056
LCC test	0.7064±0.0646	0.3833±0.1353	0.8255±0.0624	0.3876±0.1153	0.3744±0.1178
SCC val	0.6551±0.0153	0.5638±0.0490	0.6763±0.0367	0.5122±0.0163	0.5319±0.0233
SCC test	0.6579±0.0471	0.5440±0.0757	0.6743±0.0817	0.5078±0.0527	0.5157±0.0470

The highest values of interest are highlighted in bold.

Table S5. 2-classes dataset, **KNN: test accuracy = 0.7725±0.0437**

	auc	tpr	tnr	ppv	fscore
ADC val	0.8244±0.0142	0.7525±0.0165	0.8084±0.0156	0.7947±0.0153	0.7728±0.0146
ADC test	0.8208±0.0350	0.7270±0.0638	0.8180±0.0356	0.7992±0.0396	0.7597±0.0499

The highest values of interest are highlighted in bold.

Table S6. 2-classes dataset harmonized, **Naïve-Bayes: test accuracy = 0.5857±0.0418**

	auc	tpr	tnr	ppv	fscore
ADC val	0.6146±0.0180	0.4640±0.0323	0.7589±0.0310	0.6247±0.0486	0.5271±0.0346
ADC test	0.5933±0.0541	0.4187±0.0645	0.7527±0.0719	0.5987±0.0768	0.4842±0.0619

The highest values of interest are highlighted in bold.
